# Metabolomics reveal root differential metabolites of different root-type alfalfa under drought stress

**DOI:** 10.3389/fpls.2024.1341826

**Published:** 2024-01-25

**Authors:** Kun Wang, Li-Li Nan, Jing Xia, Shi-Wen Wu, Li-Li Yang

**Affiliations:** Key Laboratory of Grassland Ecosystem of Ministry of Education, College of Pratacultural Science, Gansu Agricultural University, Lanzhou, China

**Keywords:** alfalfa, drought stress, differential metabolites, phytohormones, amino acids

## Abstract

**Introduction:**

Alfalfa (*Medicago sativa* L.) is the favored premium feed ingredient in animal husbandry production which is in serious jeopardy due to soil moisture shortages. It is largely unknown how different root types of alfalfa respond to arid-induced stress in terms of metabolites and phytohormones.

**Methods:**

Therefore, rhizomatous rooted *M. sativa* ‘Qingshui’ (or QS), tap-rooted *M. sativa* ‘Longdong’ (or LD), and creeping rooted *M*. *varia* ‘Gannong No. 4’ (or GN) were investigated to identify metabolites and phytohormones responses to drought conditions.

**Results:**

We found 164, 270, and 68 significantly upregulated differential metabolites were categorized into 35, 38, and 34 metabolic pathways in QS, LD, and GN within aridity stress, respectively. Amino acids, organic acids, sugars, and alkaloids were the four categories of primary differential metabolites detected, which include 6-gingerol, salicylic acid (SA), indole-3-acetic acid (IAA), gibberellin A_4_ (GA_4_), abscisic acid (ABA), trans-cinnamic acid, sucrose, L-phenylalanine, L-tyrosine, succinic acid, and nicotinic acid and so on, turns out these metabolites are essential for the resistance of three root-type alfalfa to aridity coercing.

**Discussion:**

The plant hormone signal transduction (PST) pathway was dramatically enriched after drought stress. IAA and ABA were significantly accumulated in the metabolites, indicating that they play vital roles in the response of three root types of alfalfa to water stress, and QS and LD exhibit stronger tolerance than GN under drought stress.

## 1 Introduction

Alfalfa (or Lucerne) (*Medicago sativa* L.), as a high-protein legume and more drought-resistant than other forage, is extensively grown in semi-arid and arid regions of China (Nan et al., 2019; Li et al., 2022), but still the growing drought poses a main threat to alfalfa acreage and output (Wu et al., 2017; Wasaya et al., 2021). Therefore, improving the water use efficiency and drought-resistance breeding of alfalfa are the keys to increasing alfalfa yield.

The root types of alfalfa that are currently known can be grouped into four types: tap-rooted, branch-rooted, creeping-rooted and rhizomatous-rooted types. The alfalfa root system is the main organ for absorbing soil moisture and nutrients and exploits a non-substitutable role in improving soil macroporosity. Many alfalfa varieties are tap-rooted and the gene sources come from *Medicago sativa* L. Rhizomatous-rooted, branch-rooted, and creeping-rooted alfalfa own the genes of wild *M. falcata* L. to varying degrees, with have strong resistance to drought and severe cold. Previously, numerous studies have focused on crown characteristics (Nan et al., 2012), root development ability (Nan et al., 2014), stress resistance (Nan et al., 2011), root types, and yield (Nan et al., 2012), and rhizosphere microorganisms of different root-type alfalfa (Wang et al., 2023a). However, there were few reports on the root metabolites and phytohormones of different root-type alfalfa under drought stress.

Soil moisture content is a key abiotic factor limiting plant distribution, growth, and development (Raza et al., 2023). Decreasing the rate of growth in plants, accumulating a large number of osmotic stress substances, enhancing the amount of antioxidant enzymes in the tissues, stimulating the production of secondary metabolites, and regulating gene expression have become commonly used under drought stress (Li et al., 2021; Mubarik et al., 2021; Raza et al., 2022; Raza et al., 2023). Previous research on the adaptation mechanisms of alfalfa to water stress has primarily concentrated on the above-ground part (Zhang et al., 2019b; Kang et al., 2023), nevertheless, the root system is critical for plant production (Thorup-Kristensen et al., 2020) and has just recently received more attention (Soba et al., 2019; Zhang et al., 2019a). Since there is close contact between plant roots and soil, they can sense symptoms of water scarcity and adjust their physiology, biochemistry, and structure to respond to the alterations in the surroundings (Raza et al., 2023). Low soil moisture content induces modifications in plant root metabolism at both the macro and micro levels (Raza et al., 2022). The morphology, physiology, and bioprocesses of plants are affected by water scarcity (Li et al., 2021; Mubarik et al., 2021), which can also inhibit the root of the plant absorbing nutrient elements and the regulation of relevant functional and structural genes (Epie et al., 2019).

Metabolites not only control changes in plant phenotype but also serve as a bridge between phenotype and gene relationships (Yang et al., 2019). Metabolomics can reflect changes in metabolites and reveal the reaction mechanisms of plants to stressful conditions, which is a useful technique for studying plant environmental stress (Han et al., 2023). The same is true for secondary metabolites, which are indispensable to plant signaling, defense, and safety (Savoi et al., 2016; Kumar et al., 2021; Nicolas-Espinosa et al., 2023). It has been discovered that plants accumulate more than 2×10^5^ secondary metabolites, which serve as essential to their biological growth (Wang et al., 2015; Ma et al., 2016). Furthermore, some primary metabolites (carbohydrates, amino acids, nucleic acids, and organic acids) and several secondary metabolites (alkaloids, phenolics, quinones, flavonoids, and terpenoids) are also closely related to plant adversity resistance (Ma et al., 2016; Tschaplinski et al., 2019). The production of proteins, osmoregulatory and defensive metabolites, and reactive oxygen species-scavenging systems are some of the strategies used by plants to withstand the effects of dryness (Ma et al., 2016; Mubarik et al., 2021), which is an intricate biological mechanism involving various metabolic changes (Wasaya et al., 2021). Compared to controlling genes and other metabolic pathways, metabolites may result in more adjustments in plant response to arid stress and can be more direct targets for strengthening drought tolerance in plants. Long-term insufficient water absorbed in plants caused a noticeable rise in the contents of amino acids, tricarboxylic acid cycle (TCA) metabolites, and secondary metabolites in *Pinus sylvestris* leaves (Ma et al., 2021). Osmotic stressors, such as polyol (mannitol and sorbitol), sugars, and amino acids (proline), accumulated throughout drought and showed notable variations among tea trees with varying drought tolerance (Nyarukowa et al., 2016).

Currently, we have insufficient understanding regarding the relationship between drought resistance and modifications in alfalfa metabolites. Key secondary metabolites such as IAA, GAs, ABA, and cytokinins (CTKs) participate in plant stress regulation (Marquez-Lopez et al., 2019; Raza et al., 2022). Metabolites are the basis of biological phenotype, which can improve our ability to intuitively and efficiently understand biological processes and their mechanisms. The alteration of metabolites is the most important way for plants to respond to stress, which is achieved by regulating the metabolic network and leading to the synthesis and production of certain metabolites. In recent years, mass spectrometry (LC-MS) has been acknowledged as a vital instrument (Tang et al., 2017; Huang et al., 2018; Gao et al., 2019) for the identification of various plant species, allowing the investigation of changes in metabolites due to genetic modifications and the environment (Kumar et al., 2021; Zhang et al., 2021a). In this work, we identify metabolites altered by water scarcity and investigate their participation pathways in more detail. Identifying relevant metabolic networks may help us think more broadly about the drought resistance of these plants and help us comprehend how various root-type alfalfa cope with drought.

## 2 Materials and methods

### 2.1 Plant materials and experimental conditions

The alfalfa used in the experiment consisted of rhizomatous-rooted *M. sativa* ‘Qingshui’ (or QS), tap-rooted *M. sativa* ‘Longdong’ (or LD), and creeping-rooted *M. varia* ‘Gongnong’No. 4 (or GN). Alfalfa seeds that were uniform and plump were selected and disinfected with 5% NaClO and 70% ethanol solution in sequence (20 min) and finally rinsed the remaining solution on the surface of the seeds with distilled water (3 times). Plant the seeds uniformly in sand-filled pots and grow them in a growth chamber (YSTH-B8-20, ESHENGTAIHE CTRL TECH, China). Growth conditions: 16 h light and 8 h dark cycle, maintain a relative humidity of around 60%, and 450 mol m^-2^s^-1^ of photosynthetic light flux density from May 10, 2021, to July 26, 2021. The nutrients and water required for alfalfa growth are provided through Hoagland’s nutrient solution (1 L Hoagland solution containing 945 mg·L^-1^ Ca(NO_3_)_2_·4H_2_O, 506 mg·L^-1^ KNO_3_, 80 mg·L^-1^ NH_4_NO_3_, 136 mg·L^-1^ KH_2_PO_4_, 493 mg·L^-1^ MgSO_4_·7H_2_O, 2.78 mg·L^-1^ FeSO_4_·7H_2_O, 3.73 mg·L^-1^ EDTA-Na_2_, 6.2 mg·L^-1^ HBO_3_, 8.6 mg·L^-1^ ZnSO_4_·7H_2_O, 0.025 mg·L^-1^ GuSO_4_·5H_2_O, 0.83 mg·L^-1^ KI, 22.3 mg·L^-1^ MnSO_4_·4H_2_O, 0.25 mg·L^-1^ Na_2_MoO_4_·2H_2_O, 0.025 mg·L^-1^ CoCl_2_·2H_2_O, pH=6), which is watered every two days (Hoagland and Arnon, 1950). When the seedlings reach the two-leaf stage, 20 seedlings with the same growth and uniform distribution should be retained in each pot, for a total of 36 pots.

### 2.2 Treatment of drought stress

When the average plant height of alfalfa reaches about 40 cm (July 20, 2021), dissolve PEG 6000 in the Hoagland nutrient solution and water each pot to cause osmotic stress in alfalfa. The solution can provide osmotic pressures of 0 MPa (CK, Control), -1.0 MPa (MD, Moderate Drought Stress), and -2.0 MPa (SD, Severe Drought Stress), respectively (Michel and Kaufmann, 1973).

### 2.3 Sample collection and preservation

After 7 days of stress treatment (July 26, 2021) (Wang et al., 2023a), there was a significant change in leaf morphology, with leaf edges wrinkled and some leaves turning yellow and falling off. Then, to start sample collection, first separate the alfalfa roots from the sand and rinse them with distilled water to absorb the surface moisture. Finally, different repeated root samples of the same treatment were cut and thoroughly mixed, rapidly treated with liquid nitrogen, stored at −80°C, and relevant indicators were measured in the later stage.

### 2.4 Determining the concentration of phytohormones

① 5 g of the root sample was grounded in 70% chromatography methanol and extracted for 24 h (4°C), repeated the extraction, and merged with the extraction solution (3 times). ② The methanol in the extract was fully evaporated (vacuum conditions), and the residual solution was extracted with ethyl acetate (EA) three times, finally merging the extract and evaporating the EA. ③ The extracts were dissolved again with 70% chromatography methanol (2 mL) and passed through an organic filter (0.22 μm) membrane for the determination of phytohormones by High-Performance Liquid Chromatography (Waters Arc-2998 PDA Waters, Waters Corporation, USA) (Al-Amri, 2021). Test conditions: reversed-phase column (C18); mobile phase A: methanol, mobile phase B: 0.1% phosphorus; pH=3.5, column temperature: 30°C, flow speed: 0.8 mL/min, wavelength: λ GA_3 = _239 nm, λ IAA=255 nm, λ ABA= 208 nm, λ (ZT) = 254 nm.

### 2.5 Metabolite extraction

① 100 mg of liquid nitrogen-milled samples, put them in 2.0 mL tubes, and add 500 μL of 80% methanol; ② vortex and shake, let it take an ice bath (5 min), and centrifuge it at 12000 rcf (4°C, 20 min); ③ take the supernatant and dilute it with ultrapure water to a methanol content of 53%; ④ collect the supernatant (12000 rcf, 4°C, 20 min), and analyzed by LC-MS (Kumar et al., 2021). Chromatographic conditions: chromatographic column-HypesilGoldcolumn (C18), column temperature: 40°C, flow rate: 0.2 mL/min; mobile phase A: 0.1% formic acid, mobile phase B: methanol; pH 9.0. UHPLC-MS/MS analyses were performed using a Vanquish UHPLC system (Thermo Fisher, Germany).

### 2.6 Statistical analysis

The detected metabolites were annotated using the KEGG (https://www.genome.jp/kegg/pathway.html). The metabolomics data was processed using the software metaX (Wen et al., 2017) and subjected to principal component analysis (PCA) and partial least squares discriminant analysis (PLS-DA). Calculated the statistical significance (*P*-value) based on the *t*-test and the multiplicity of different Fold Change (FC) of two groups. Criteria for screening: VIP > 1, *P* < 0.05, and FC ≥ 2.

## 3 Results

### 3.1 Metabolomic data quality assessment

**Figure 1:**
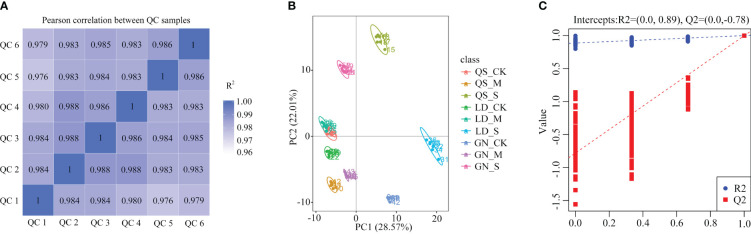
Sample correlation analysis **(A)**, sample PCA analysis **(B)** and PLS-DA model plots **(C)**. QC, Quality Control. PC, Principal component. Partial Least Squares Discrimination Analysis (PLS-DA) is a supervised statistical method for discriminant analysis, the method uses partial least squares regression to model the relationship between tabolite expression and sample category to achieve the prediction of sample category, the PLS-DA model of each comparison group was established, and the model evaluation parameters (R2, Q2) obtained by 7-fold cross-validation (seven cycles of crossvalidation, when the number of biological replicates of the samples n<=3, it is k cycles of cross-validation, k=2n), if R2 and Q2 are closer to 1, it indicates that the model is more stable and reliable. QS, rhizomatous-rooted *Medicago sativa* ‘Qingshui’; LD, tap-rooted *M. sativa* ‘Longdong’; GN, creeping-rooted *M.varia* Martin ‘Gongnong No.4’. CK, Control; M, Medium stress; S, Severe stress.

The higher the correlation of Quality Control (QC) samples (R^2^ closer to 1) indicates higher the quality of the obtained raw data. Every sample has an R^2^ value above 0.97, which is extremely close to 1 (**Figure 1A**), indicating that the data are reliable. The six sets of samples from the same experimental treatments under different water stresses clustered together, demonstrating powerful repeatability among the experimental treatments (**Figure 1B**). Partial Least Squares Discrimination Analysis (PLS-DA) was performed to identify the metabolites that responded to drought stress. R2 and Q2 of each treatment were close to 1, which indicated that the date is robust and accurate, and the subsequent analysis may be performed (**Figure 1C**).

### 3.2 Principal component analysis

**Figure 2:**
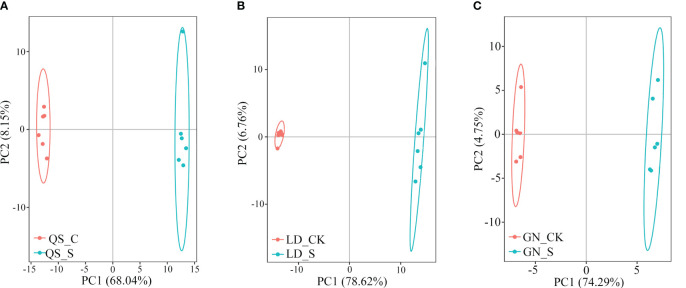
Principal component analysis of samples of QS **(A)**, LD **(B)** and GN **(C)** alfalfa under severe stress compared to CK. PC1, Principal component 1; PC2, Principal component 2. QS, rhizomatous-rooted *Medicago sativa* ‘Qingshui’; LD, tap-rooted *M. sativa *‘Longdong’; GN, creeping rooted *M.varia* Martin ‘Gongnong No.4’. CK, Control; S, Severe stress.

Drought stress causes significant changes in the metabolic products of three types of root-type alfalfa. The PC1 of QS, LD, and GN (68.04%, 78.62%, and 74.29%) significantly separated the control treatment (CK) from severe drought (SD) (**Figure 2**). On the score map, individual samples were remarkably far off from one another, indicating obvious variations between samples but not between groups. The PC1 of LD and GN root metabolism in response to drought was more than the variance contribution of QS, demonstrating that the metabolic changes in LD and GN were more significant compared to those in QS under water deficit stress.

### 3.3 Screening of differentially expressed metabolites

**Figure 3:**
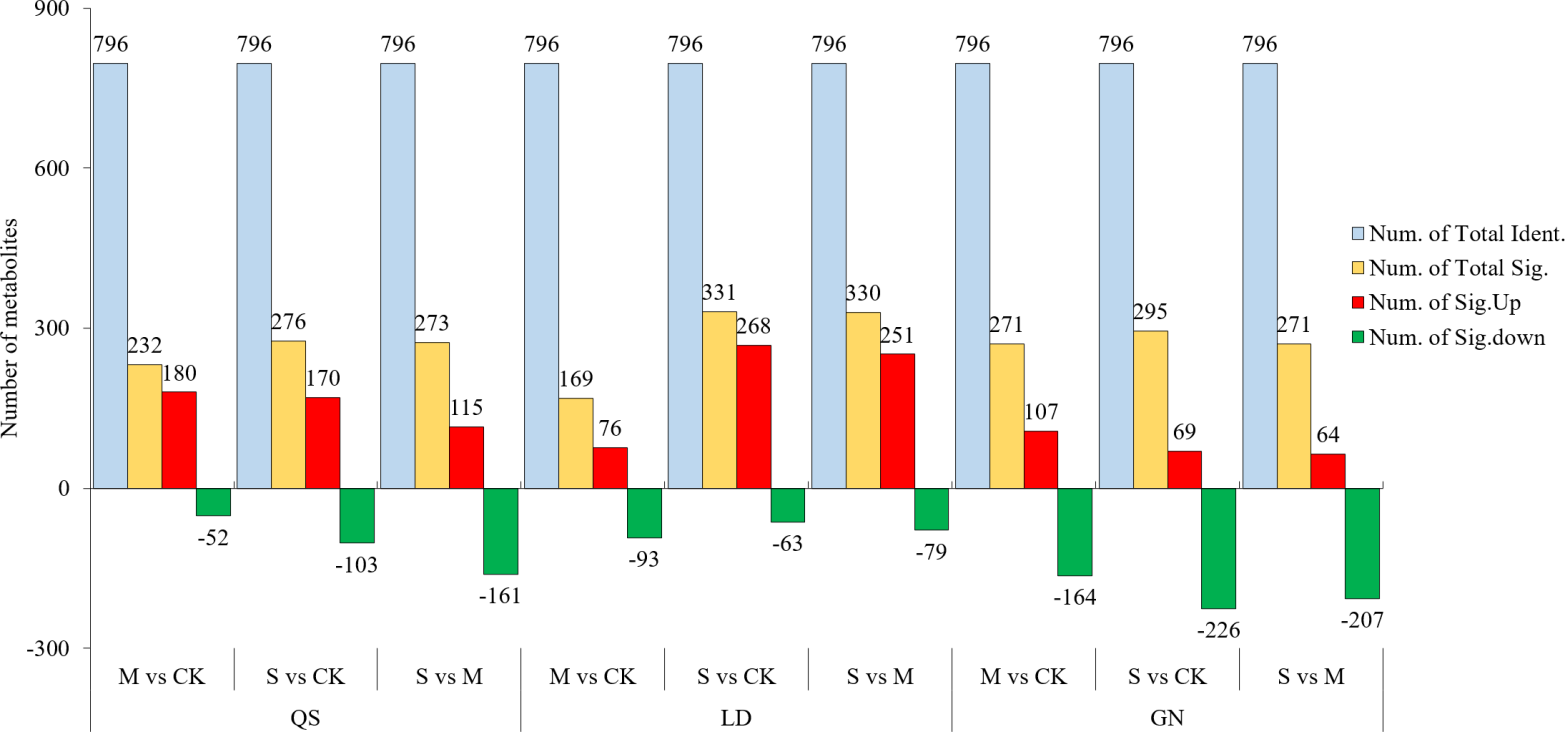
Metabolite statistics of QS, LD and GN alfalfa under drought stress. QS, rhizomatous-rooted *Medicago sativa* ‘Qingshui’; LD, tap-rooted *M.sativa* ‘Longdong’; GN, creeping-rooted *M.varia* Martin ‘Gongnong No.4’. CK, Control; M, Medium stress; S, Severe stress.

**Figure 4:**
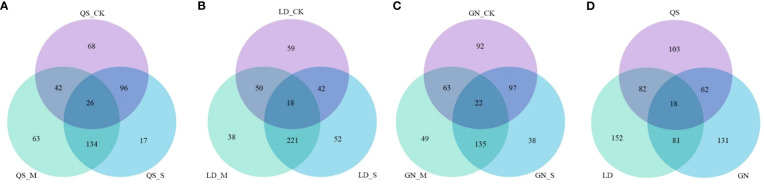
Venn plot of differential metabolites unique to QS **(A)**, LD **(B)**, and GN **(C)** and coexisting metabolites **(D)** among the three root types of alfalfa after drought stress. QS, rhizomatous-rooted *Medicago sativa* ‘Qingshui’; LD, tap-rooted *M.sativa* ‘Longdong’; GN, creeping-rooted *M.varia* Martin ‘Gongnong No.4’. CK, Control; M, Medium stress; S, Severe stress.

Combining the *t*-test and the PLS-DA method (VIP value) to screen for DEMs. An amount of 796 metabolites were found in three root-type alfalfa under drought conditions (**Figure 3**). QS, LD, and GN were found to have 265, 333, and 292 DEMs compared to each CK treatment under SD stress. There were 164, 270, and 68 upregulated DEMs, and 101, 63, and 224 downregulated DEMs, respectively. The majority of DEMs were upregulated in QS and LD, which accounted for 61.89% and 81.08% of the total number of metabolites. LD had a greater number of DEMs upregulated as compared to QS and GN. The number of DEMs unique to QS, LD, and GN were 26, 18, and 22 at various degrees of water stress, respectively (**Figure 4**). The three root-type alfalfa shared 18 species of DEMs under SD stress (**Figure 4D**), whereas LD and GN had much more special metabolites than QS, with 152, 131, and 103, respectively. This suggests that QS has an excellent ability to adapt to water deficits and drought-stress conditions with less metabolite modification.

### 3.4 Analysis of differential metabolites

**Figure 5:**
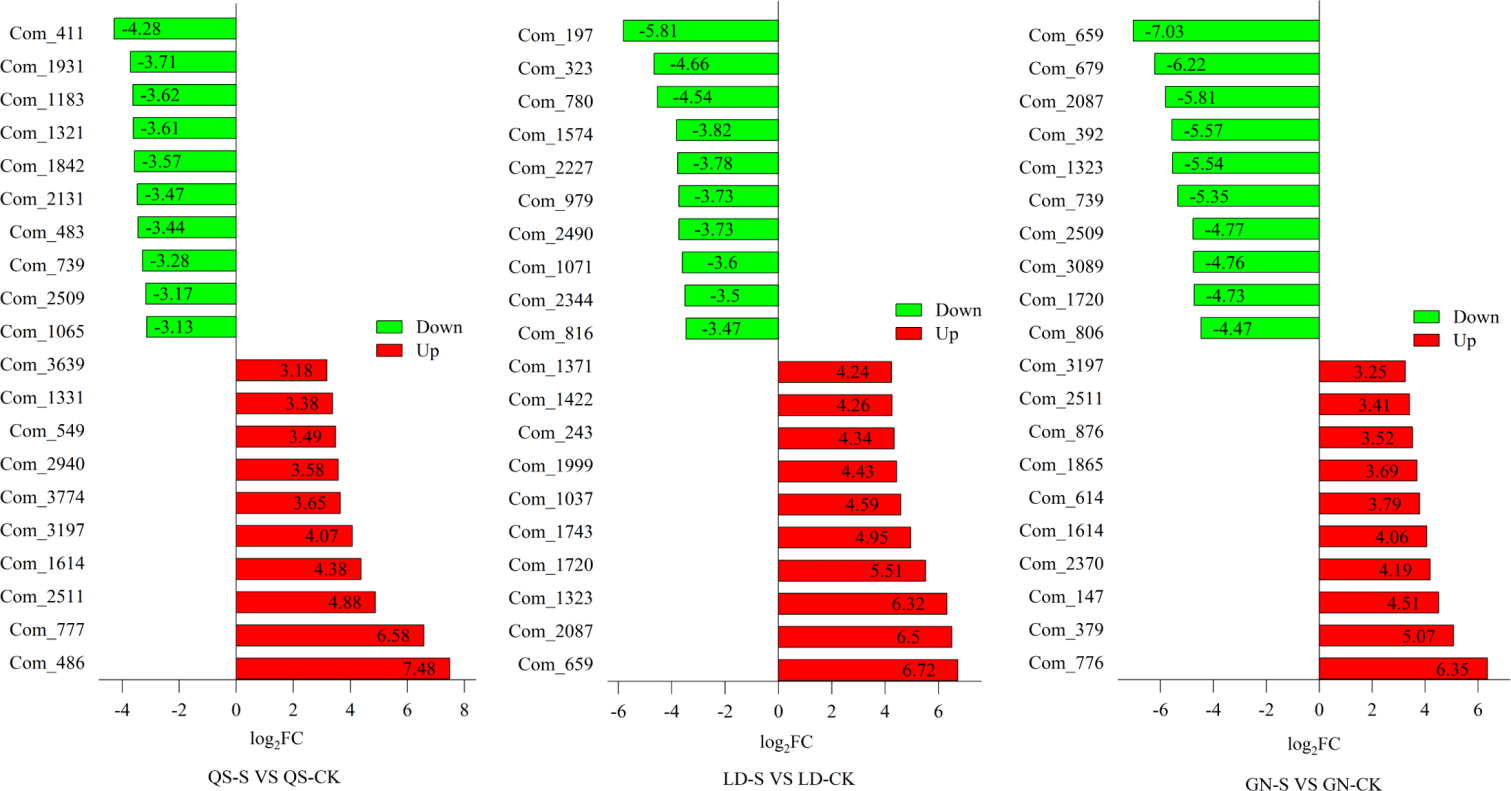
Major metabolite statistics for QS, LD and GN alfalfa top 10 after severe drought stress. FC, Fold Change. QS, rhizomatous-rooted *Medicago sativa* ‘Qingshui’; LD, tap-rooted *M. sativa* ‘Longdong’; GN, creeping rooted *M.varia* Martin ‘Gongnong No.4’. CK, Control; M, Medium stress; S, Severe stress.

The top 20 (upregulated and downregulated) DEMs from the Fold Change (FC) plots of QS, LD, and GN (**Figure 5**). Illustrate that the metabolites changed dramatically to response aridity coercing and that the same metabolites are present in three root-type alfalfa simultaneously. For instance, metabolite codenames (Com) Com_1614 and Com_2511 simultaneously appeared in QS and GN and all of them showed an upregulated trend. Among the downregulated metabolites Com_739 and Com_2509 simultaneously appeared in QS and GN. As an example, Com_1720, Com_1323, Com_2087, and Com_659 were upregulated in LD alfalfa, while the opposite was true in the GN. This showed that the same varieties of metabolites play various metabolic roles in different alfalfa varieties.

### 3.5 Functional annotation and enrichment analysis of the differential metabolite KEGG

**Figure 6:**
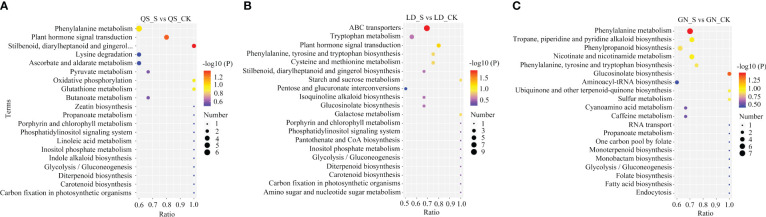
KEGG analysis of major metabolic pathways in QS **(A)**, LD **(B)**, and GN **(C)** alfalfa after severe drought stress. QS, rhizomatous-rooted *Medicago sativa* ‘Qingshui’; LD, tap-rooted *M.sativa* ‘Longdong’; GN, creeping rooted *M.varia* Martin ‘Gongnong No.4’. CK, Control; M, Medium stress; S, Severe stress.

According to the KEGG database (**Figure 6**; **Table 1**), Based on the *P*-value of metabolic pathways, select the top three significantly enriched metabolic pathways in three root types of alfalfa. QS, LD, and GN have 35, 38, and 34 differential metabolites annotated into the first three metabolic pathways, respectively. DEMs (13) including 6-gingero, SA, IAA, GA_4_, ABA, and others were dramatically enriched of stilbenoid-diarylheptanoid gingerol biosynthesis (SDG) (*P ≤* 0.04), PST (*P ≤* 0.05), and phenylalanine metabolism (PM) (*P ≤* 0.10) under SD stress in QS (**Table 1**). The DEMs (15) of sucrose, L-phenylalanine, galacturonic acid, IAA, GA_4_, ABA, and others were discovered of ABC-transporters (*P ≤* 0.01), PST (*P ≤* 0.06), and galactose metabolism (GM) (*P ≤* 0.13) in LD (**Table 1**). The *P*-values of the SDG, PST, and ABC-transporters metabolic pathways in QS and LD were all less than 0.05, indicating that they play a crucial role in the resistance of both alfalfa varieties to drought stress. DEMs (15) including SA, L-tyrosine, L-phenylalanine, succinic acid, and others significantly annotated in GN’s metabolic pathways of PM (*P ≤* 0.03), glucosinolate biosynthesis (*P ≤* 0.04), and nicotinate-nicotinamide metabolism (*P ≤* 0.10) (**Table 1**). The *P*-values of PM and glucose biosynthesis metabolic pathways are both less than 0.05, indicating that they are key metabolic pathways for GN alfalfa to cope with drought-stress environments. This shows that coping with water deficit stress is closely tied to these metabolic pathways and metabolites, especially the remarkably enriched ABA and SA in three root-type alfalfa, demonstrating that they are crucial to how alfalfa responds to drought.

**Table 1:** The number of metabolites and metabolic pathways to the top 3.

	Metabolic pathway	*P*	Metabolites
QS	stilbenoid-diarylheptanoid and gingerol biosynthesis	0.04	6-gingerol, piceatannol, chlorogenic acid
	plant hormone signal transduction	0.05	salicylic acid, indole-3-acetic acid, gibberellin A_4_, abscisic acid
	phenylalanine metabolism	0.10	salicylic acid, succinic acid, 3-hydroxyphenylacetic acid, trans-cinnamic acid, phenylacetaldehyde, fumaric acid
LD	ABC transporters	0.01	sucrose, L-phenylalanine, galacturonic acid, biotin, betaine, L-cystine, inositol, glutathione
	plant hormone signal transduction	0.06	indole-3-acetic acid, gibberellin A_4_, abscisic acid, jasmonic acid
	galactose metabolism	0.13	sucrose, inositol
GN	phenylalanine metabolism	0.03	salicylic acid, L-tyrosine, L-phenylalanine, succinic acid, hippuric acid, trans-cinnamic acid, phenylacetaldehyde
	glucosinolate biosynthesis	0.04	L-tyrosine, L-phenylalanine, methionine
	nicotinate and nicotinamide metabolism	0.10	nicotinuric acid, succinic acid, 1-methylnicotinamide, quinolinic acid

QS, rhizomatous-rooted *Medicago sativa* ‘Qingshui’; LD, tap-rooted *M. sativa* ‘Longdong’; GN, creeping-rooted *M.varia* Martin ‘Gongnong No.4’.

**Figure 7:**
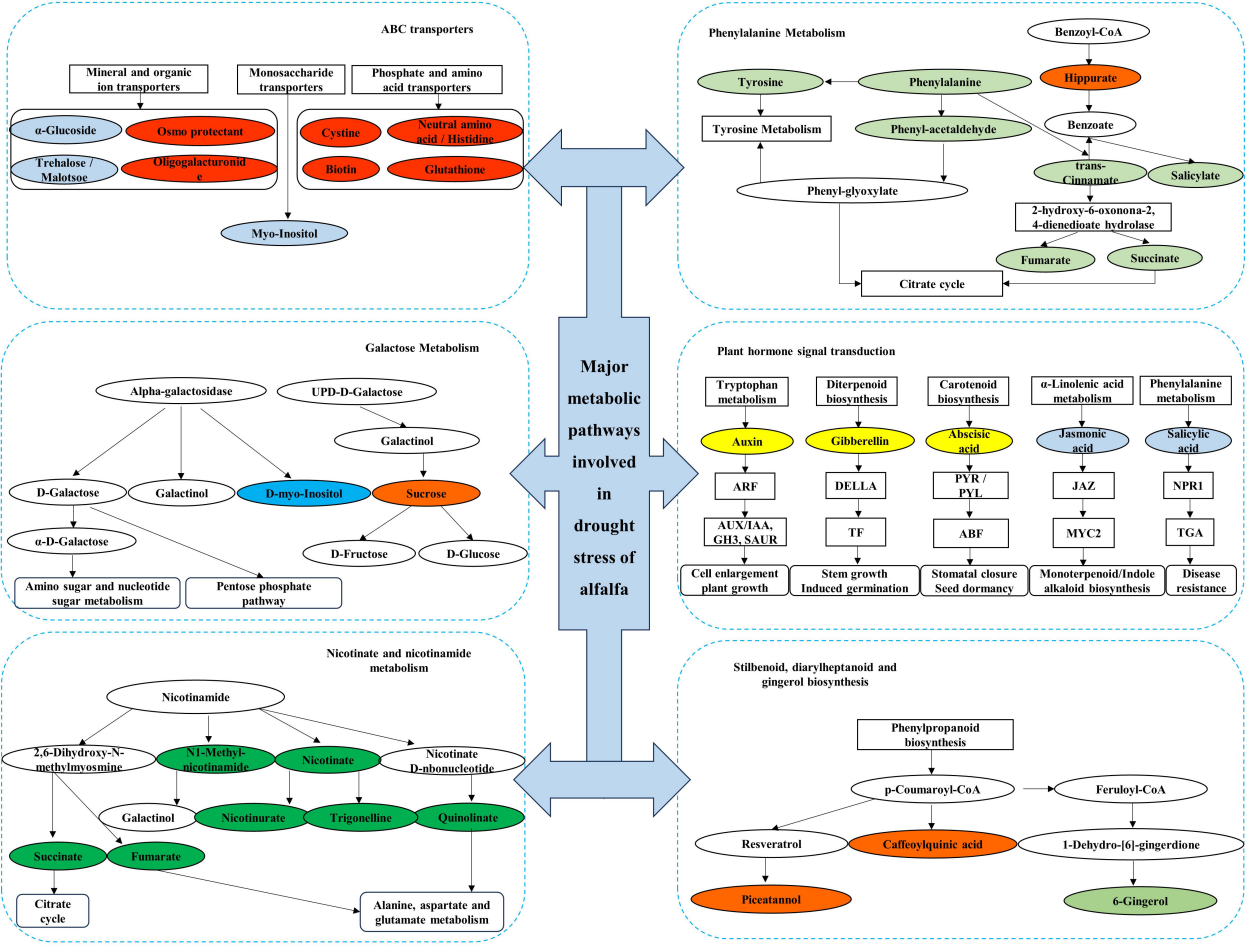
Major metabolic pathways involved in drought stress of alfalfa. Boxes in the figure indicate metabolic pathways annotated to drought stress, and ellipses indicate metabolites annotated to drought stress. Green ellipses indicate metabolites down-regulated under relatively severe drought stress in QS and GN, and orange indicates metabolites up-regulated under relatively severe drought stress in the GN; yellow indicates metabolites upregulated under relatively severe drought stress in LD and QS, and light blue indicates metabolites down-regulated under relatively severe drought stress in LD and QS; blue indicates metabolites down-regulated under relatively severe drought stress in LD, and red indicates metabolites upregulated under relatively severe drought stress in LD; and dark green indicates metabolites downregulated under relatively severe drought stress in the GN. QS, rhizomatous-rooted *Medicago sativa* ‘Qingshui’; LD, tap-rooted *M.sativa* ‘Longdong’; GN, creeping rooted *M.varia* Martin ‘Gongnong No.4’. CK, Control; M, Medium stress; S, Severe stress.

The top 10 metabolic pathways in QS, LD, and GN were annotated for 28, 31, and 36 metabolites, respectively, and the metabolic pathways mainly classification were SDG, PST, ABC transporters, GM, PM, nicotinate-nicotinamide metabolism (NM) (**Figure 7**). Up-regulated metabolites account for 28.57%, 83.87%, and 5.6% of the total metabolites, respectively, mainly distributed in the metabolic pathways of tryptophan metabolism (TM), SDG, lysine degradation, starch-sucrose metabolism (SM), PST, and PM (**Table 2**). The fact that the DEMs annotated in LD and GN are much more abundant than QS alfalfa reveals that QS alfalfa can withstand drought stress with fewer metabolite changes.

**Table 2:** Number of metabolic pathways annotated to differential metabolites.

Number	Metabolic pathway	Number/species of annotated differential metabolites obtained		
		QS	LD	GN
1	ubiquinone and other terpenoid-quinone biosynthesis	0	0	2
2	tryptophan metabolism	0	5	0
3	tropane, piperidine and pyridine alkaloid biosynthesis	0	0	5
4	Sulfur metabolism	0	0	2
5	stilbenoid, diarylheptanoid and gingerol biosynthesis	3	2	0
6	starch and sucrose metabolism	0	2	0
7	propanoate metabolism	2	0	0
8	plant hormone signal transduction	4	4	0
9	phenylalanine biosynthesis	0	0	5
10	phenylalanine, tyrosine and tryptophan biosynthesis	0	3	3
11	phenylalanine metabolism	6	0	7
12	oxidative phosphorylation	2	0	0
13	nicotinate and nicotinamide metabolism	0	0	5
14	lysine degradation	3	0	0
15	isoquinoline alkaloid biosynthesis	0	2	0
16	glycolysis/gluconeogenesis	1	0	0
17	glutathione metabolism	2	0	0
18	glucosinolate biosynthesis	0	2	3
19	galactose metabolism	0	2	0
20	cyanoamino acid metabolism	0	0	2
21	caffeine metabolism	0	0	2
22	butanoate metabolism	2	0	0
23	ascorbate and aldarate metabolism	3	0	0
24	ABC transporters	0	9	0
Total	total number of differential metabolites annotated	28	31	36
	total number of metabolic pathways annotated	10	9	10

QS, rhizomatous-rooted *Medicago sativa* ‘Qingshui’; LD, tap-rooted *M.sativa* ‘Longdong’; GN, creeping-rooted *M.varia* Martin ‘Gongnong No.4’.

### 3.6 Effects of drought stress on phytohormone contents in alfalfa root

**Figure 8:**
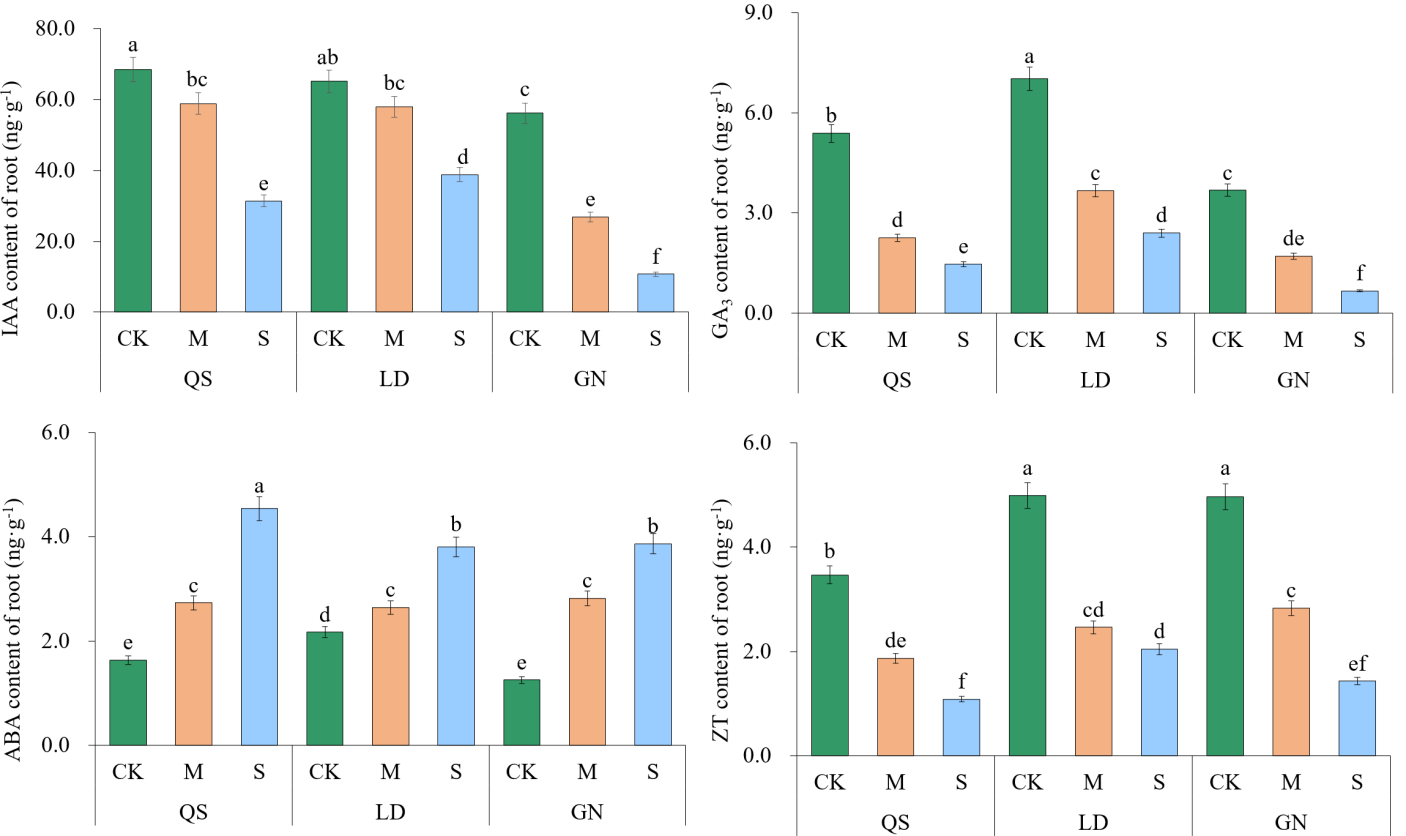
Effect of root endogenous hormone contents of alfalfa under drought stress. Different lowercase letters between treatments indicate significant differences (*P*<0.05). QS, rhizomatous-rooted *Medicago sativa* ‘Qingshui’; LD, tap-rooted *M. sativa* ‘Longdong’; GN, creeping-rooted *M.varia* Martin‘Gongnong No.4’. CK, Control; M, Medium stress; S, Severe stress.

Except for ABA, IAA, GA_3_, and ZT, all tended to decrease as the degree of stress increased (**Figure 8**). The IAA, GA_3_, and ZT contents of QS, LD, and GN were considerably lower than those of CK under SD stress, with QS decreasing by 54.13%, 72.76%, and 68.76%; LD decreased by 40.37%, 65.78%, and 58.89%; GN decreased by 80.94%, 81.77%, and 71.14%, respectively. The fact that QS and LD experienced less decline than GN states clearly that they were more drought-resistant and drought-tolerant than GN. The levels of ABA in the three varieties of alfalfa roots exhibited an increasing trend with increasing stress, rising by 177.87%, 75.25%, and 208.89%, respectively. When compared to GN, QS and LD showed lower increases, which demonstrates that they were more capable of enduring the stress of drought than GN.

## 4 Discussion

Osmoregulation is one of the strategies for plants to cope with water deficit stress (Wasaya et al., 2021). High sucrose and sugar alcohol accumulation enhanced plant tolerance and contributed to scavenging free radicals generated by aridity stress (Skalska et al., 2021). The glycolysis or gluconeogenesis pathways were measurably enriched in QS, LD, and GN under SD stress in this study, and arbutin as a natural antioxidant was involved in these pathways were all upregulated, the results show that these pathways are crucial for water deficit. SA, succinic acid, 3-hydroxyphenylacetic acid, trans-cinnamic acid, phenylacetaldehyde, and fumaric acid of QS and GN were all down-regulated in the PM pathway, and one study found that the accumulation of organic acids improves the capacity of *Plumeria rubra* to withstand water shortage conditions (Sun et al., 2023). Research has found that the increase in ABA content under drought stress inhibits the expression of *GmPAL1*, which is the first rate-limiting enzyme encoding the PM pathway, thereby affecting downstream products such as SA and flavonoids (La et al., 2023). SA serves as a signaling molecule to control gene expression to affect physiological properties (Gilani et al., 2020) and enhance the drought resistance of plants (Mubarik et al., 2021; Raza et al., 2023). Succinic acid is a critically important component of the TCA in plants, which can affect their respiration rates and increase their tolerance (Yue et al., 2018; Hijaz and Killiny, 2019), its significant enrichment in QS and GN indicates a positive effect on drought response.

In the phenylalanine-tyrosine and tryptophan biosynthesis pathway, the metabolites of L-phenylalanine, erythrose-4-phosphate, L-tyrosine, and indole were altered but enriched in LD and GN in opposite ways, suggesting that the pathway responds to drought stress in different ways. It was found that tea apoptosis induces high expression of genes related to the synthesis of endogenous hormones, thus affecting changes in their contents (ABA, JA, SA), and that ABA regulates phenylalanine, tyrosine, and tryptophan biosynthesis and phenylalanine metabolism by affecting theanine (Xu et al., 2020). This indicates that changes in ABA levels are in correlation with metabolic pathways, have the ability to control metabolic pathways, and may have an impact on the production of related metabolites. Inositol, the primary metabolite of QS and LD, was decreased significantly during the process of the inositol phosphate metabolism and the phosphoinositide signaling pathway, which mainly manifested as response and adaptation to abiotic stimuli such as cold and arid conditions (Tan et al., 2013). Other studies have found that SA stimulates the expression of genes involved in phosphatidylinositol synthesis in *Arabidopsis*, hence affecting inositol production (Gulabani et al., 2022).

Amino acids are essential osmoregulators for plants under abiotic stress. Research has shown that amino acids can induce stomatal closure and reduce water evaporation under drought stress (Sakata et al., 2023). The tryptophan metabolic pathway was dramatically enriched in LD in this study. Tryptophan and isoleucine indicated that significantly increase with water deficit stress (Ignjatovic-Micic et al., 2015; Kong et al., 2022). However, it has also been demonstrated that only drought-tolerant cultivars have higher tryptophan levels (Bao et al., 2023), and tryptophan metabolites can enhance plant stress resistance under adverse conditions (Hasnain et al., 2023). ABC transporter proteins have certain drought-resistant capabilities under drought stress and provide triacylglycerol to glycerol kinase (Han et al., 2022), which then stimulates the synthesis of sucrose (Sharma and Nayyar, 2014). The accumulation of linoleic acid content can promote the oxidation of fatty acids, and its oxidation products can provide energy for plant growth and enhance the ability of stress tolerance in plants (Goepfert and Poirier, 2007).

Phytohormone content in plants is significantly altered by drought stress due to its impact on phytohormone metabolism (Ghafari et al., 2020; Talaat, 2023). The PST pathway responds simultaneously to water deficit stress in three alfalfa varieties, and there is a close correlation between hormone synthesis and metabolic pathways (Wang et al., 2023b). The trend of IAA and GA_4_ is contrary to the enrichment tendency shown by the metabolites of SA and jasmonic acid (JA) in the PST pathway. IAA can promote cell division, elongation, differentiation, and the formation of new organs (Shani et al., 2017), as well as enhance plant adaptability to abiotic stresses by maintaining antioxidant enzyme activity in plant cells (Khan et al., 2021). Reduced IAA levels due to drought stress restrict plant growth. Reduced GA_4_ content can improve plant drought resistance (Kang et al., 2011). The growth-promoting hormones of IAA, GA_3_, and ZT with low content can slow down plant growth to cope with water deficit. The inhibition hormone of ABA with high content can promote stomatal closure to reduce transpiration loss and promote root water absorption to improve the ability of drought resistance (Zhang et al., 2021b). ABA maintains the growth of the root system under water deficit conditions (Zhang et al., 2020) and promotes the transportation of assimilates to the reservoir (Chen et al., 2020; Zhang et al., 2021b), additionally, phytohormones crosstalk with each other to improve resistance to environment stress (Raza et al., 2023). In this research, the content of three growth-promoting hormones in alfalfa tends to decrease, revealing that the growth is limited by drought. In contrast, the content of growth-inhibiting hormone (ABA) increased with the intensification of drought stress in three root types of alfalfa, which further confirms that ABA can improve plant adaptability to drought stress.

## 5 Conclusions

Drought stress affects the metabolic pathways and changes in metabolites in alfalfa roots. The main metabolic pathways in three root-type alfalfa are SDG, PST, ABC transporters, PM, NM, and GM. For QS, LD, and GN, the metabolites include amino acids, organic acids, sugars, and alkaloids, mainly involved in osmotic pressure regulation, signal transduction, and ABC transporter proteins. With increasing drought stress, the levels of IAA, GA_3_, and ZT were decreased, while ABA content increased in roots. Overall, QS and LD had more resistance than GN under drought stress.

## Data availability statement

The raw data supporting the conclusions of this article will be made available by the authors, without undue reservation.

## Author contributions

KW: Writing – original draft. LN: Methodology, Writing – review & editing, Data curation. JX: Data curation, Writing – review & editing. SW: Formal analysis, Writing – review & editing. LY: Software, Writing – review & editing.
